# Feasibility of extracting usable DNA from blood samples stored up to 21 years in the DiPiS study

**DOI:** 10.1038/s41598-025-08257-y

**Published:** 2025-07-15

**Authors:** Agnes Andersson Svärd, Ellen Viberg, India von Platen, Ida Jönsson, Markus Lundgren, on behalf of the DiPiS study group, A. Ramelius, C. Andersson, R. Bennet, M. Ask, J. Bremer, C. Brundin, C. Cilio, H. Elding Larsson, C. Hansson, G. Hansson, S. Ivarsson, B. Jonsdottir, I. Jonsson, B. Lindberg, B. Lernmark, Å. Lernmark, J. Melin, M. Lundgren, A. Carlsson, E. Cedervall, B. Jönsson, K. Larsson, J. Neiderud

**Affiliations:** 1 Department of Clinical Sciences, Lund University, Lund, Sweden; 2 Department of Pediatrics, Kristianstad Hospital, Kristianstad, Sweden; 3 Department of Paediatrics, Ängelholm Hospital, Ängelholm, Sweden; 4 Department of Paediatrics, Ystad Hospital, Ystad, Sweden; 5 Department of Paediatrics, Helsingborg Hospital, Helsingborg, Sweden; 6 Department of Pediatrics, Skåne University Hospital, Kristianstad, Sweden

**Keywords:** Blood sample, Long-term sample storage, DNA isolation, DNA concentration, DNA quality, Biological techniques, Cell biology, Molecular biology, Endocrinology, Medical research

## Abstract

This study assesses the feasibility of extracting high-quality DNA from blood samples stored at – 20 °C for up to 21 years under suboptimal conditions. It addresses sample mishandling in research, where many samples lack proper biobank protocols. Prior studies focused on short-term storage and controlled conditions, highlighting the negative effects of freeze–thaw cycles. This study evaluates whether DNA from long-term stored samples under suboptimal conditions can still meet quality standards for research purposes. Genomic DNA was extracted from 1012 capillary blood samples from the Diabetes Prediction in Skåne study. Samples were stored at – 20 °C for 7–21 years, and DNA was isolated using QIAamp DNA Blood Mini kits. DNA quantity, purity, and quality were analysed using spectrophotometry and automated electrophoresis. Overall, 75.7% of samples met quality standards for DNA quantity (≥ 20 ng/µL) and purity (A260/280 ratio 1.7–1.9), with the highest proportion in 12-year samples (83.5%). DNA quality was further assessed in 270 samples, where 57.8% had a DNA Integrity Number (DIN) of 7 or higher. This study suggests that historical blood samples stored under suboptmal conditions can still be viable for modern genomic analyses.

## Introduction

Whole blood is a common source of high quantity and quality DNA for molecular applications due to its relative ease of acquisition^1^. Blood samples are routinely collected in research studies and biobanking efforts across the globe, including for clinical and epidemiological studies, that can be, or have been, stored for years. DNA yield is directly correlated to the blood volume available for DNA isolation^2^. Many factors can affect the quality of DNA isolated from blood samples, such as collection method, type of collection tube, thawing and re-freezing, transportation, and storage time and facilities^3^. Several studies have examined the effects of different storage methods on blood sample condition when used^4–6^, and storage temperature and duration affect DNA quality and yield^4,6,7^. Provided that the correct blood collection tubes have been used, blood samples stored at 4 °C for a short period will still yield DNA of acceptable quality^8,9^; indeed, high concentrations of satisfactory quality DNA can be isolated from blood samples stored for eight weeks at 4 °C or below in the presence of EDTA or heparin^6^. Overall, however, the desired temperature for long-term storage of whole blood samples destined for DNA isolation is − 80 °C^3^. Whole blood samples can also be frozen at − 20 °C long-term^3,10,11^, but DNA yields tend to be lower at this temperature^8,11^. Di Pietro et al. showed that whole blood samples stored long-term at − 20 °C yielded high-quality DNA for genotyping studies^12^, while Chen et al. concluded that storing blood samples long-term for between two and 19 years at − 30 °C did not affect the quantity and quality of DNA^13^.

Nevertheless, unforeseen events occurring during long-term storage of study samples, such as malfunctioning storage facilities or freezers or repeated freeze–thaw cycles, might affect sample quality and hence the DNA yield and quality. Few studies have investigated DNA isolated from samples stored long-term that have been heavily mistreated through thawing and freezing. Long-term storage is a necessity when samples are not immediately processed and analysed, so it is important to determine the effects of long-term storage on DNA yield and quality^14^.

Here we analyse a unique cohort of samples mistreated while in long-term storage. In the Diabetes Prediction in Skåne (DiPiS) study, children donated a capillary blood sample for autoantibody analysis annually. Throughout the study, plasma was removed and stored separately from, but adjacent to, its corresponding blood cells. The blood cell samples were thawed and refrozen an unknown number of times when plasma samples were used for autoantibody analyses, but the blood cell samples have yet to be used in any analysis. This set of 7500 blood cell samples could potentially be incredibly valuable scientifically as they are part of the DiPiS cohort, representing a considerable amount of work, dedication, and time from both families and staff participating in the study over many years.

Therefore, the aim of this study was to determine whether DNA of satisfactory quality could be isolated from capillary blood samples in long-term storage in DiPiS. DNA obtained from old samples and samples stored under less-than-optimal conditions could provide invaluable data for studies with large sample collections in long-term storage.

## Methods

### Study samples

DiPiS was a prospective, population-based cohort study that investigated the genetic and environmental factors contributing to, or triggering, the development of type 1 diabetes. Between September 2000 and August 2004, newborns in Skåne were screened for risk of developing type 1 diabetes. Children at increased risk of type 1 diabetes were invited to participate in follow-up from two to 15 years of age or until diabetes onset, whichever occurred first^15^.

During follow-up, the children donated capillary blood samples for autoantibody analyses each year. If the children developed two or more autoantibodies, they were followed four times per year and had their sample drawn by a study nurse.

For each annual follow-up, a blood sample test kit containing a 0.5 mL EDTA capillary blood collection test tubes (BD, Franklin Lakes, NJ) were sent to the participant, and the blood was drawn either by their primary health care provider or by the parents at home. The sample was sent to the laboratory by post at room temperature. The time between blood draw and arrival at the laboratory could be up to seven days but was usually between one and three days.

Once a blood sample arrived at the laboratory, the plasma was separated from the blood in the capillary EDTA tube. Samples were placed in boxes in the order they arrived at the laboratory and stored in multiple – 20 °C freezers at our research facilities. The blood cells have been stored for up to 21 years and have been thawed an unknown number of times due to malfunctioning storage facilities (such as freezers breaking down). There is no kept record of when or which freezer has broken down over the years. Sample boxes have been moved between freezers and physically taken out of the freezers for autoantibody analyses as blood cell samples are stored adjacent to its corresponding plasma sample. Boxes physically removed from the freezers when selecting samples for autoantibody analyses subjected the blood cell samples to room temperature and freeze–thaw cycles, due to low sample volumes that thaw easily. Boxes that remained in the freezers were subjected to relatively minor insults related to opening the freezer and finding samples in different boxes.

Of the 7500 blood samples saved in DiPiS, 1012 samples were randomly selected for evaluation in the current study. 478/1012 (47.2%) were from females. The samples were randomly selected from different boxes for each of the five time periods during the DiPiS follow-up: late 2002 to early 2003, 2007, 2011, 2015, and 2016. From each time period, 200 samples were randomly selected, except for 2016 where 212 samples were selected.

### DNA isolation

DNA was isolated from thawed samples using QIAamp DNA Blood Mini Kits (Qiagen, Hilden, Germany) according to the manufacturer’s instructions^16^, with minor modifications. Briefly, the QIAamp DNA Blood Mini Kit protocol was optimized using fresh whole blood collected in EDTA tubes, from which plasma was removed prior to overnight storage at – 20 °C. The volume of the study samples varied from < 10 µL to 500 µL. As reagent volumes used in the protocol are based on sample volume, samples with volumes < 250 µL were diluted to 250 µL with phosphate-buffered saline (PBS) prior to adding Qiagen protease and lysis buffer to ensure consistency with the study samples. To ensure no wastage and optimum yield, the entire blood volume in a tube was used for DNA extraction. If a sample volume was > 250 µL, the sample was loaded onto the column in two steps. The sample mixture was first transferred from the EDTA tube to the microcentrifuge tube by pipette but was later poured into the microcentrifuge instead to minimize sample loss and unnecessary use of pipette tips. However, for some samples, it was difficult to avoid transferring large clots to the column. Too much starting material and insufficient lysis could clog the filter and significantly affect the DNA yield^16^. Using a pipette, we avoided large clots, which improved the elution step and avoided clogging the filter, which could impact elution and decrease DNA quantity. The sample was mixed with Qiagen protease and lysis buffer by pulse vortexing (Vortex-genie 2, Scientific Industries, Bohemia, NY) for 15 s. This way, any dried blood and any remaining small clots were eventually dissolved in the lysis buffer through vortexing.

Protease and lysis buffer were added directly into the 0.5 mL EDTA tube, instead of first transferring the sample to a microcentrifuge tube, to minimize the risk of leaving residual dried blood in the tube. The isolated DNA was stored at – 20 °C.

### DNA analysis and quantification

DNA was quantified by applying 2 μl of each sample to a NanoQuant Plate and reading on the Tecan Infinite 200 Pro instrument (Tecan, Männedorf, Switzerland). Raw absorbance data were obtained at 260, 280, and 310 nm, and blanked absorbance data were obtained at 260 and 280 nm. DNA concentration (ng/µL) and A260/280 ratio were calculated using i-control^™^ Microplate Reader Software (i-control 2.0, Tecan).

An Agilent 2200 TapeStation (Agilent Technologies, Santa Clara, CA) was used for quality control and to evaluate DNA fragmentation in 270 of the DNA samples selected randomly but evenly from the five time periods. All samples with DNA concentrations > 100 ng/μL were diluted in elution buffer to a concentration between 10 and 100 ng/μL. Samples were prepared and analysed according to instructions (Genomic DNA ScreenTape Assay, Agilent Technologies). Gel pictures, DNA sizing, and calculation of DNA integrity number (DIN) were performed by the TapeStation Analysis Software (A.02.01, Agilent Technologies).

DNA quality was evaluated from each sample based on the % integrated area around the bp peak. The peaks and integrated area were automatically detected and set during analysis and manually checked during data analysis. The software algorithm classifies DNA by the DIN, which is scaled 1–10 based on the level of degradation, where 1 is the most degraded and 10 is the least degraded^17^. A DIN > 7 is accepted as high molecular weight DNA. In an Agilent study, a sample with DIN 9.2 and fragment size of 53,929 bp is seen as intact^17^.

### Statistical analyses

Descriptive statistics were compiled by years of storage. Medians and inter-quartile ranges were calculated for DNA concentrations, A260/280 and DNA yields, blood volume available per sample, and DNA yield per milliliter of blood. Medians were compiled as the data were not normally distributed.

DNA quantity and quality were investigated in groups defined by the five time periods from which the samples originated (2002–2003, 2007, 2011, 2015, and 2016). The acceptability criteria of sufficient quantity, purity and quality for DNA samples included a concentration of at least 20 ng/µL to ensure sufficient template for PCR amplification, a purity indicated by an A260/280 ratio within the satisfactory range of 1.7–1.9, and a DNA Integrity Number (DIN) of 7 or higher to confirm minimal degradation and high genomic quality.

A DNA concentration of 20 ng/µL or higher was deemed satisfactory, ensuring adequate template for PCR amplification and avoiding non-specific products^18^. This aligns with Thermo Fisher and Qiagen’s recommended range of 10–100 ng/µL for optimal PCR performance^19,20^. DNA yield was calculated by multiplying the DNA concentration (μg/mL) by total sample volume (mL).

An A260/280 ratio between 1.7 and 1.9 is considered satisfactory as it indicates a balanced level of protein and nucleic acid contamination in the DNA sample. This range suggests that the DNA is relatively pure, with minimal interference from proteins or other contaminants, which is crucial for reliable performance in downstream applications such as PCR and sequencing^21,22^.

The A260/280 ratio obtained from NanoQuant was used to create three groups as a measurement of DNA quality: low (< 1.7), satisfactory (1.7–1.9), and high (> 1.9).

A DIN of 7 or higher was deemed satisfactory, indicating high genomic DNA quality with minimal degradation and maintained structural integrity. Kong et al.^17^ note that this DIN threshold, as measured by the Agilent 2200 TapeStation System, ensures fewer fragmentation events and reliable performance for sequencing and PCR applications.

Data of fragment size from TapeStation was cleaned from background noise. Background noise was identified in peaks with fragment size (< 3000 bp) and integrated area (< 2). The fragment size (bp) received from the TapeStation analysis was stratified into six groups: 8000–10,000, 10,000–20,000, 20,000–30,000, 30,000–40,000, 40,000–50,000, and 50,000–60,000 bp. Due to an error with one of the TapeStation chips, 17 samples from 2007 and 2011 were excluded prior to statistical analysis.

Histograms were used to examine the distribution of DNA concentration, ratio, fragment size, and DIN. Boxplots were used to investigate concentration, A260/280 ratio, DIN, and yield in the five time periods. Kruskal–Wallis and Wilcoxon’s tests were used to estimate the significance of differences between groups. Nominal p values < 0.005 were considered suggestive of an association. Presented p values remained significant after adjustment for multiple comparisons. R (v4.2.0, The R Foundation, Vienna, Austria; http://www.r-project.org) was used for data compilation, processing, and analysis.

## Results

### DNA quantity

**Fig. 1:**
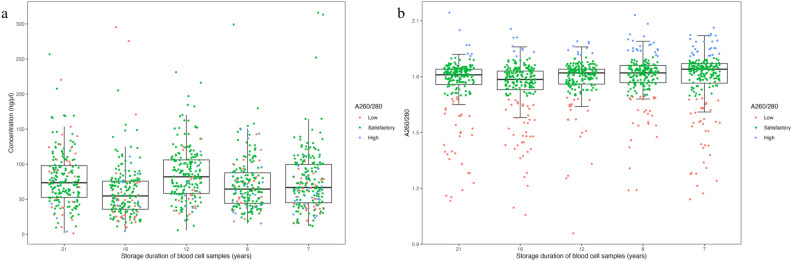
DNA concentration (ng/μL) (**a**) and A260/280 ratio (**b**) of 1012 DNA samples in the DiPiS study stratified by the years they were obtained. The colours (red, green, blue) corresponds to a low ratio (< 1.7), ratio within satisfactory range (1.7–1.9) and high ratio (> 1.9), respectively. In (**b**), an outlier with ratio 2.4 has been removed from the sample age of 7 years. Kruskal–Wallis test (p_global_ = 1.2e^-06^) suggest a significant difference between the groups. Pair-wise comparison of ratio, with samples stored for 7 years as reference showed a significant difference with samples stored for 21 (p_adj_ = 0.001), 16 (p_adj_ = 5.32e-06) and 12 (p_adj_ = 0.043) years, respectively. Concentrations and A260/280 ratios were measured by NanoQuant Plate in a Tecan Infinite 200 Pro reader.

DNA was isolated from 1012 blood cell samples from five time periods during the DiPiS follow-up period and had been stored for between seven and 21 years. DNA concentrations (ng/μL) and A260/280 ratios were acceptable in samples obtained at all five time periods (Fig. 1 and Table 1a). The DNA concentration ranged from 1.5 to 316.1 ng/μL, with a median value of 68.7 ng/μL, and 981 (96.9%) samples had an acceptable concentration equal to or greater than 20 ng/μL. The lowest and highest concentrations were observed in samples stored for 21 and 7 years, respectively. The highest median concentration was in samples stored for 12 years, while samples stored for 16 years had the lowest median concentration.

**Table 1:** Characteristics of DNA concentration and A260/280 ratio. a) DNA concentration and A260/280 ratio corresponding to storage duration of blood cell samples and b) DNA concentration stratified by A260/280 ratio distribution. The A260/280 ratio is grouped into low ratio (1.9). Concentrations and A260/280 ratios were measured using NanoQuant Plate in a Tecan Infinite 200 Pro reader.

a				Concentration (ng/µL)	A260/280			
	Sample year	Storage duration (years)	Sample size	Median [IQR]	Median [IQR]	Low (< 1.7), n (%)	Satisfactory (1.7–1.9), n (%)	High (> 1.9), n (%)
	2002–2003	21	200	73.5 [52.7, 98.0]	1.81 [1.8, 1.8]	32 (16.0)	161 (80.5)	7 (3.5)
	2007	16	200	54.7 [35.8, 75.8]	1.78 [1.7, 1.8]	38 (19.0)	153 (76.5)	9 (4.5)
	2011	12	200	82.0 [58.3, 106.1]	1.82 [1.8, 1.8]	20 (10.0)	170 (85.0)	10 (5.0)
	2015	8	200	64.5 [44.2, 87.7]	1.82 [1.8, 1.7]	26 (13.0)	153 (76.5)	21 (10.5)
	2016	7	212	66.8 [45.0, 99.6]	1.84 [1.8, 1.9]	36 (17.0)	148 (69.8)	28 (13.2)
	Overall	7–21	1012	68.7 [46.0, 93.9]	1.81 [1.8, 1.9]	152 (15.0)	785 (77.6)	75 (7.4)

b		A260/280						
		Low (< 1.7)		Satisfactory (1.7–1.9)		High (> 1.9)		Overall
	Concentration (ng/µL)	n	Med [IQR]	n	Med [IQR]	n	Med [IQR]	n (%)
	< 10	1	5.8 [3.6;7.9]	2	7.7 [6.8;8.6]	2	4.2 [4.0;4.3]	5 (0.5)
	10–19	10	18.2 [16.3;18.9]	17	16.5 [13.1;18.7]	2	16.4 [15.9;16.9]	29 (2.9)
	20–79	102	48.6 [36.6;63.2]	454	57.3 [40.5;68.5]	56	50.2 [41.5;61.7]	612 (60.5)
	80–119	23	91.8 [87.0;103.6]	223	96.7 [88.4;106.8]	11	97.1 [90.7;105.7]	257 (25.4)
	120–150	11	124.8 [122.2;138.0]	56	127.8 [124.4;138.4]	4	145.1 [142.0;147.3]	71 (7.0)
	> 150	5	220.2 [170.8;275.5]	33	168.5 [158.0;205.2]	0	NA	38 (3.8)
	All	152	54.5 [37.4;81.3]	785	72.3 [49.5;97.9]	75	55.6 [41.5;74.2]	1012 (100)

**Fig. 2:**
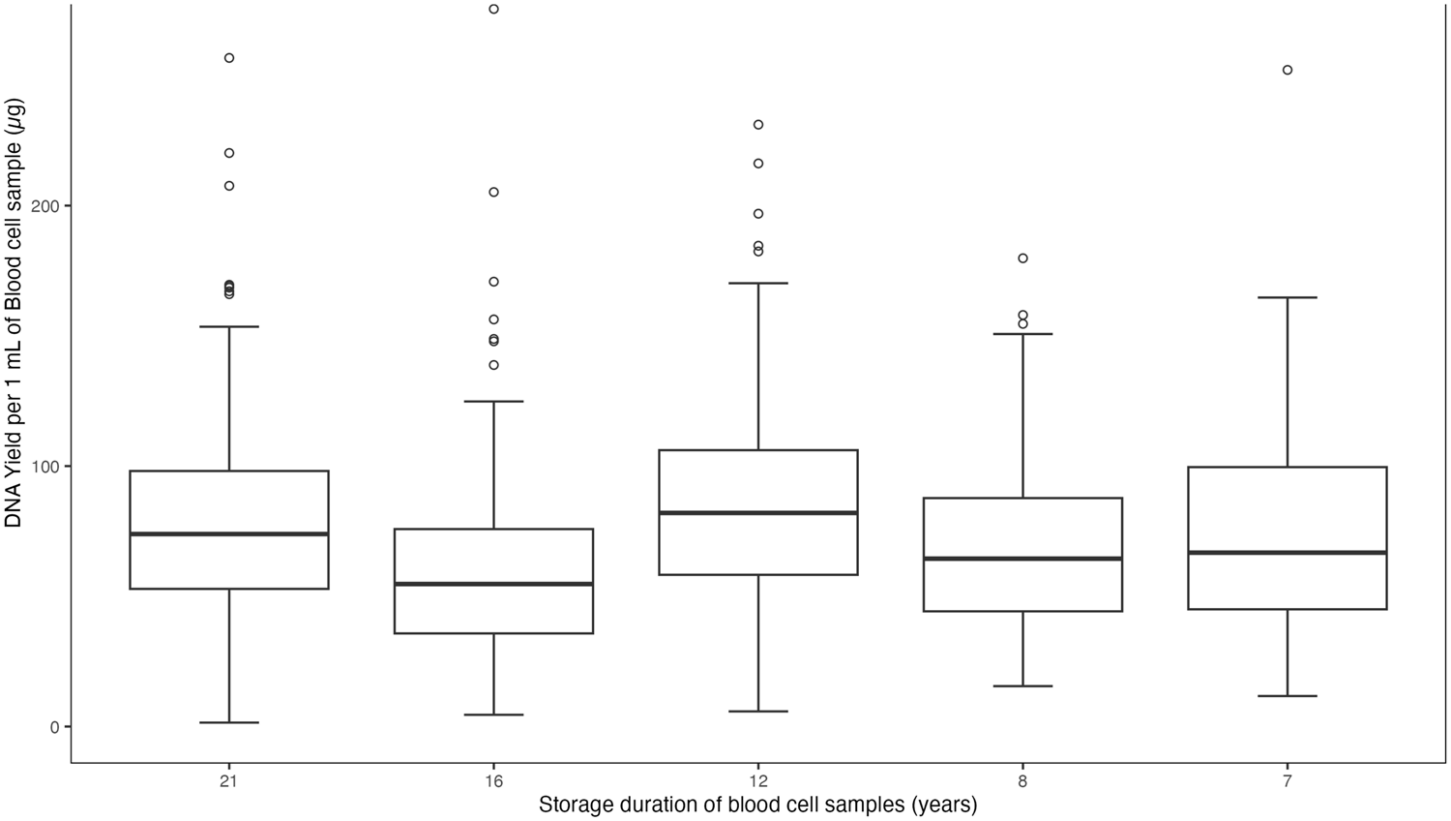
DNA yield (µg) per 1 mL of blood cell sample. Range and distribution of DNA yields per 1 mL of blood cells isolated from frozen blood cell samples stored over 7–21 years at -20 °C during which they have been heavily mistreated. In pair-wise comparisons with the samples stored for 7 years as reference, a statistically significant difference in yield was observed in samples stored for 12 and 16 years. DNA yields were calculated from concentrations measured by NanoQuant Plate in a Tecan Infinite 200 Pro reader.

DNA concentration measurements from NanoQuant Plates in a Tecan Infinite 200 Pro reader were used to calculate DNA yields (Fig. 2 and Table 2). DNA yields of samples per storage duration clustered tightly for 7 to 21 years, except for a few outliers (Fig. 2). In a pair-wise comparison with samples stored for 7 years as reference, a statistically significant difference in yield was observed in samples stored for 12 and 16 years. In addition, yield was investigated in relation to the number of times a sample was loaded onto the column, once or twice, depending on if the sample volume was less than or greater than 250 μL. A statistically significant difference in yield was observed in each of the five time periods (Supporting Fig. 3). In total, 734 (72.5%) samples had a volume less than 250 µL, while 278 (27.5%) had more than 250 µL and had to be loaded twice onto the column. An increased yield was observed for the group of samples loaded twice onto the columns.

**Table 2:** Descriptive statistics of yields.

Sample year	Storage duration (years)	Sample size	Mean (SD) blood cell volume (mL)	Mean (SD) DNA concentration (ng/µL) per sample	Median [IQR] DNA yield (µg) per sample	Mean (SD) DNA yield (µg) per sample	Mean (SD) DNA yield/mL of blood cells (µg)
2002–2003	21	200*	0.18 (0.068)	78.90 (39.00)	13.73 [8.38, 20.50]	15.40 (10.30)	79.30 (38.80)
2007	16	200	0.24 (0.059)	61.30 (38.80)	11.71 [7.82, 18.95]	14.40 (10.20)	61.30 (38.80)
2011	12	200	0.24 (0.059)	85.10 (37.80)	19.20 [13.77, 25.51]	20.60 (10.90)	85.10 (37.80)
2015	8	200	0.27 (0.060)	70.70 (36.20)	17.01 [11.62, 23.98]	19.60 (13.40)	70.70 (36.20)
2016	7	212	0.25 (0.063)	74.70 (42.20)	17.06 [10.31, 25.30]	19.40 (12.70)	74.70 (42.20)
Overall	7–21	1012	0.24 (0.068)	74.10 (39.60)	15.90 [9.87, 23.05]	17.9 (11.80)	74.20 (39.60)

DNA yields were calculated from concentrations measured by NanoQuant Plate in a Tecan Infinite 200 Pro reader.

*One sample, completely dried-in, was removed during in calculations of yield per mL blood cell sample as initial sample volume was 0 µL.

### DNA purity

The A260/280 ratio ranged from 1.0 to 2.4, with a median of 1.8, with a satisfactory ratio (1.7–1.9) obtained in 77.6% of samples (Table 1a). In total, 152 (15.0%) had a low ratio (< 1.7, range: 0.96–1.7), 785 (77.6%) had a satisfactory ratio (1.7–1.9), and 75 (7.4%) had a high ratio (> 1.9, range 1.9–2.4). The median ratio was the same for all sample groups. The percentage of samples with a high ratio decreased with increasing time of storage. Most samples with a ratio > 1.9 were very close to being satisfactory, with only a few samples having a much higher ratio.

We next investigated DNA concentrations stratified by ratio (Table 1b). Supporting Fig. 1 show the distribution of DNA concentration and A260/280 ratio. Overall, most samples had either a concentration of 20–79 ng/μL (612, 60.5%) or 80–119 ng/μL (257, 25.4%). Only 5 (0.5%) had a concentration of less than 10 ng/μL and 38 (3.8%) had a concentration greater than 150 ng/μL. In all groups of low, satisfactory or high ratio, most samples had 20–79 ng/μL followed by 80–119 ng/μL. The least number of samples in each group had either a concentration lower than 10 or greater than 150 (Table 3).

**Table 3:** Characteristics of DNA samples with a concentration above 20 ng/µL and a satisfactory or high A260/280 ratio corresponding to sample age.

			Concentration (ng/µL)	A260/280		
Sample year	Storage duration (years)	Sample size	Median [IQR]	Median [IQR]	Satisfactory (1.7–1.9), n (%)	High (> 1.9), n (%)
2002–2003	21	164	72.9 [55.8, 96.3]	1.82 [1.79, 1.84]	158 (96.3)	6 (3.7)
2007	16	156	61.7 [40.7, 77.8]	1.8 [1.77, 1.84]	148 (94.9)	8 (5.1)
2011	12	177	85.3 [61.7, 106.7]	1.82 [1.78, 1.85]	167 (94.4)	10 (5.6)
2015	8	171	64.9 [45.4, 88.3]	1.83 [1.8, 1.86]	151 (88.3)	20 (11.7)
2016	7	169	72.3 [52.4, 102.3]	1.85 [1.81, 1.87]	142 (84.0)	27 (16.0)
Overall	7–21	837	71.8 [50.8, 96.8]	1.83 [1.79, 1.85]	766 (91.5)	71 (8.5)

The A260/280 ratio is grouped into a low ratio (< 1.7), a ratio within the satisfactory range (1.7–1.9) and a high ratio (> 1.9). Concentrations and A260/280 ratios were measured using NanoQuant Plate in a Tecan Infinite 200 Pro reader.

DNA concentrations and A260/280 ratios stratified by storage duration are presented in Supporting Table 1. Pair-wise comparisons of ratios, with samples stored for 7 years (one outlier with ratio 2.4 removed) as the reference, showed no significant difference with samples stored for 8 years but significant differences for samples stored for 21 (p_adj_ = 0.001), 16 (p_adj_ = 5.32e^-06^), and 12 (p_adj_ = 0.043) years, respectively. An acceptable DNA concentration of at least 20 ng/μL and with either a satisfactory or high A260/280 ratio was observed for 837 (82.7%) samples (Table 3).

Samples where a clot had been removed during DNA isolation had mostly a satisfactory ratio, but were also present in the low and high ratio groups, with a DNA concentration mostly ranging between 20 and 119 ng/µL, but also present in 10–19 ng/µL and 120–150 ng/µL (data not shown).

### DNA quality

**Fig. 3:**
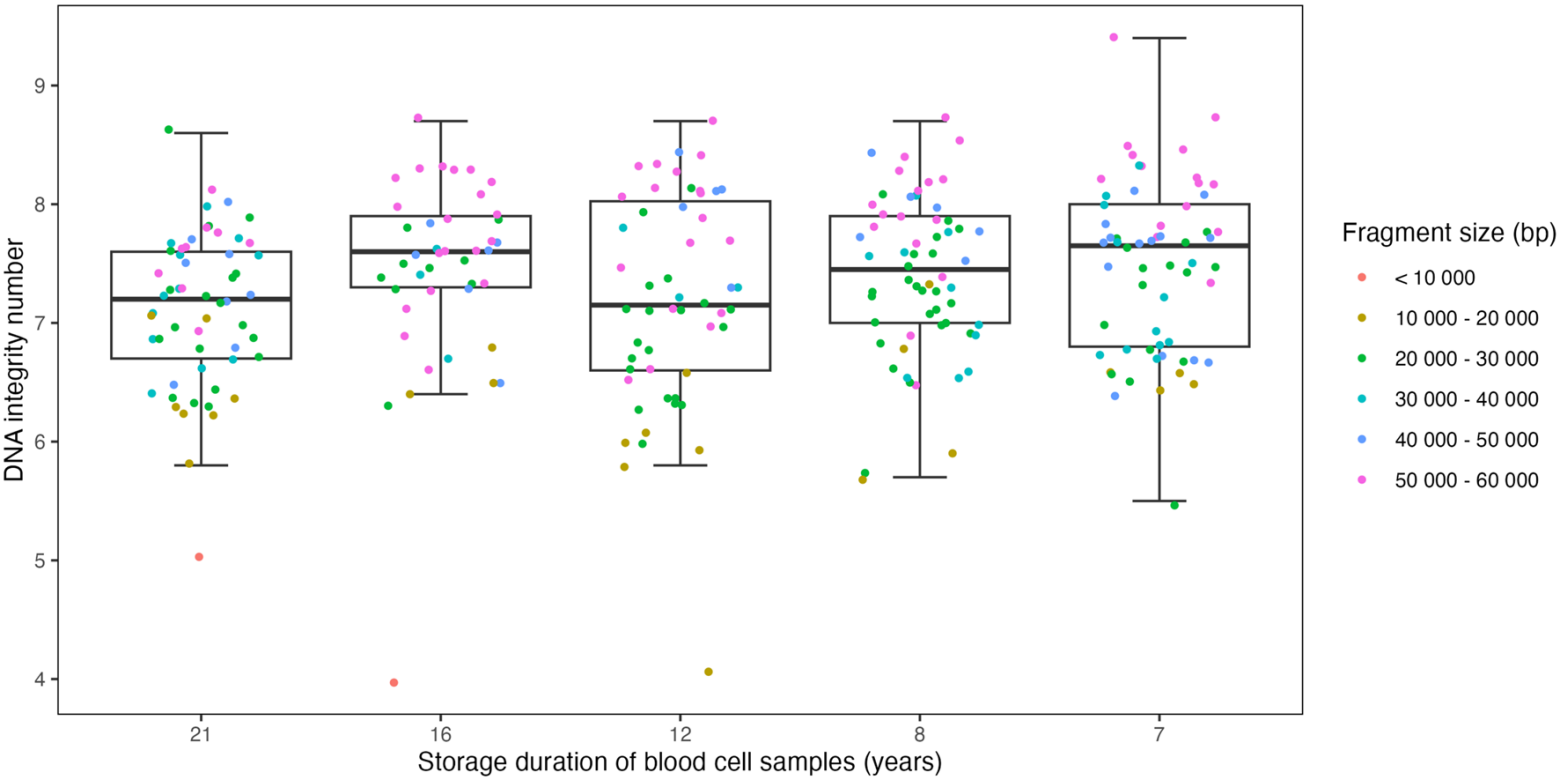
DNA integrity number (DIN) measured in base pairs (bp), stratified by storage duration and colored by fragment size. Outliers with a DIN value below 5.1 have been removed. Kruskal–Wallis test (p_global_ = 0.032) suggest a significant difference between the groups. Pair-wise comparison with samples stored for 7 years as reference showed significant difference with samples stored for 21 years (p_adj_ = 0.031). DIN’s and fragment sizes were measured by Agilent 2200 TapeStation.

DNA fragmentation was investigated in 270 (26.7%) samples randomly and evenly selected from the five sample age groups. DIN and fragment size were visualized across the five time periods (Fig. 3). Fragment size was divided into three groups (8000–19,999, 20,000–39,999, and 40,000–60,000 bp). Most samples had a fragment size over 40,000 bp, and only two samples had a fragment size below 10,000 bp. Five samples had a fragment size > 60,000 bp, which is the maximum fragment size measured by the TapeStation instrument. Pair-wise comparisons with samples stored for 7 years as reference showed significant differences with samples stored for 21 years (p_adj_ = 0.031). A DIN equal to or greater than 7 was observed for 189 (70%) samples (median 7.4; Table 4). The median DIN was lowest for samples aged 12 and 21 years. Overall, the integrated area was large for all samples with a median of 97.3%, minimum of 41.6%, and maximum of 100%.

**Table 4:** Characteristics of DNA integrity number (DIN) and fragment size, measured in base pairs (bp), stratified by storage duration.

		DIN			Size (bp)			
Storage duration (years)	Sample size	Median [IQR]	< 7, n (%)	7–10, n (%)	Median [IQR]	8,000–19,999, n (%)	20,000–39,999, n (%)	40,000–60,000, n (%)
21 years	56	7.2 [6.2, 7.6]	21 (37.5)	35 (62.5)	30,892 [22026, 44850]	8 (14.3%)	31 (55.4%)	17 (30.4%)
16 years	42	7.6 [7.3, 7.9]	9 (21.4)	33 (78.6)	49,605 [28916, 53024]	4 (9.5%)	12 (28.6%)	26 (61.9%)
12 years	52	7.2 [6.6, 8.0]	18 (34.6)	34 (65.4)	30,861.5 [23520, 52945]	6 (11.5%)	23 (44.2%)	23 (44.2%)
8 years	60	7.5 [7.0, 7.9]	14 (23.3)	46 (76.7)	31,403.5 [26076, 50334]	4 (6.7%)	34 (56.7%)	22 (36.7%)
7 years	60	7.7 [6.8, 8.0]	19 (31.7)	41 (68.3)	39,472.5 [26921, 49922]	4 (6.7%)	27 (45.0%)	29 (48.3%)
All	270	7.4 [6.8, 7.8]	81 (30.0)	189 (70.0)	35,577 [25508, 51093]	26 (9.6)	127 (47.0)	117 (43.3)

The fragment size is grouped into 8 000–19 999 bp, 20 000–39 999 bp and 40 000–60 000 bp. Outliers with a DIN value below 5.1 have been removed. DIN’s and fragment sizes were measured by Agilent 2200 TapeStation.

Shorter fragments (up to 20,000 bp) generally have a lower DIN than longer fragments (Fig. 3). The largest median fragment size was observed in 16-year-old samples, while the shortest fragments were observed in 21-year-old samples (2002–2003). The youngest 7-year-old samples had the highest percentage of long fragments (between 40,000 and 60,000 bp), while 21-year-old samples had the lowest percentage of long fragments.

### Evaluation of DNA quality and integrity across storage durations

The proportions of samples meeting the predefined acceptability criteria for quantity, purity, and quality are summarized in Table 5. Overall, 75.7% of the 1,012 samples met the criteria for quantity (≥ 20 ng/µL) and purity (A260/280 ratio of 1.7–1.9) (Table 5a). The highest proportion of acceptable samples was found in those stored for 12 years (83.5%), while the lowest was in samples stored for 7 years (67.0%). Among the 270 samples analysed with the TapeStation, 57.8% met the additional criterion of an acceptable DNA Integrity Number (DIN) of 7 or higher (Table 5b). The highest proportion of these samples was found in those stored for 8 years (61.7%), and the lowest in those stored for 7 years (55.0%).

**Table 5:** Sample proportions meeting predefined acceptability criteria of sufficient quantity and purity and quality. The predefined acceptability criteria include a DNA concentration ≥ 20 ng/µL and satisfactory A260/280 ratios (1.7–1.9) in a) all samples and b) 270 samples with the additional quality measurement from Tapestation with an acceptable DNA integrity number (DIN) of 7 or higher.

a	Sample year	Storage duration (years)	Sample size	Concentration ≥ 20 ng/µL, n (%)	Satisfactory A260/280 (1.7–1.9), n (%)	All predefined criteria
	2002–2003	21	200	194 (97.0)	161 (80.5)	158 (79.0)
	2007	16	200	189 (94.5)	153 (76.5)	148 (74.0)
	2011	12	200	197 (98.5)	170 (85.0)	167 (83.5)
	2015	8	200	196 (98.0)	153 (76.5)	151 (75.5)
	2016	7	212	202 (95.3)	148 (69.8)	142 (67.0)
	Overall	7–21	1012	978 (96.6)	785 (77.6)	766 (75.7)

b	Sample year	Storage duration (years)	Sample size	Concentration ≥ 20 ng/µL, n (%)	Satisfactory A260/280 (1.7–1.9), n (%)	DIN ≥ 7 n (%)	All predefined criteria
	2002–2003	21	56	55 (98.2)	51 (91.1)	35 (62.5)	32 (57.1)
	2007	16	42	40 (95.2)	32 (76.2)	33 (78.6)	24 (57.1)
	2011	12	52	51 (98.1)	48 (92.3)	34 (65.4)	30 (57.7)
	2015	8	60	59 (98.3)	48 (80.0)	46 (76. 7)	37 (61. 7)
	2016	7	60	60 (100.0)	49 (81. 7)	41 (68.3)	33 (55.0)
	Overall	7–21	270	265 (98.1)	228 (84.4)	189 (70.0)	156 (57.8)

## Discussion

This is arguably not only a paper about sample “mistreatment” but also represents a "real-world" scenario seen in hundreds of research labs around the world where samples (not limited to blood but other biofluids and tissues) are kept as parts of historical research cohorts under less-than-ideal conditions outside formal biobanks with their protocols and governance structures. Times have changed and there is greater awareness of the importance of quality biobanking. Most DNA quality studies have simulated storage conditions and handling. Studies on DNA quality have examined the impact of storage conditions and repeated freeze–thaw cycles. Shao et al.^23^ found that repeated freeze-thawing negatively affects DNA stability, leading to degradation, especially after multiple cycles. Similarly, Safarikova et al.^24^ assessed the effect of different storage conditions on DNA integrity, concentration, and purity. Both studies emphasize that preserving DNA under optimal conditions is essential for maintaining its integrity and quality, crucial for subsequent analyses like PCR or sequencing.

This study was undertaken to determine the fate of 7500 blood cell samples stored at – 20 °C for seven to 21 years under less-than-optimal conditions. We investigated the feasibility, quantity, and quality of DNA extraction from 1012 samples. As the capillary blood cell samples were stored adjacent to their corresponding plasma samples, they had been mistreated through an unknown number of freeze–thaw cycles as study analyses have progressed in DiPiS. The concern was that the samples would be too damaged from this mistreatment and that the samples would have to be discarded, prompting investigation of whether they were fit for future analyses. Genomic DNA was isolated from 1012 blood cell samples stored for 7–21 years using QIAamp DNA Blood Mini Kits.

The results from our study are unique from previous findings as we investigate mistreated samples stored long-term. Previous studies have investigated DNA isolated from blood samples stored for up to a few months in the freezer^4–6^, at different temperatures^13^, or the quality of DNA stored for a long time under controlled forms in a biobank^25^. Hara et al. investigated storage conditions of forensic blood samples and blood stains, and concluded that storage below – 20 °C was required to prevent DNA degradation during long-term (20-year) storage^26^. We found that it is possible to isolate satisfactory quality DNA from samples mistreated through repeated freeze–thaw cycles. There are no records of which sample boxes that were or were not physically removed from storage and subjected to room temperature and freeze–thaw cycles once or multiple time. Therefore, it was not possible to analyse the DNA integrity by grouping the samples based on the degree mishandling they were subjected to over the years.

The median A260/280 ratios were ~ 1.8 across all years of collection, suggesting high DNA purity regardless of the duration of storage. We did not measure the A260/230 ratio, so we could not evaluate the presence of other organic residues. However, any organic residues would likely represent artifacts resulting from the inadequate removal of organic chemicals used in the extraction chemistry and not directly associated with the sample itself.

DNA was successfully isolated, relative to the acceptability criteria of ≥ 20 ng/μL, from almost all 978 (96.6%) samples, as 29 (2.9) had a concentration of 10–19 ng/μL and only 5 (0.5%) had a concentration below 10 ng/μL. The A260/280 ratio was satisfactory (1.7–1.9) for 785/1012 (77.6%) samples, indicating that a majority of samples are of satisfactory quality. However, other studies^27,28^ classify DNA with a ratio between 1.7 and 2.0 as pure. Applying the wider ratio range to this study, only 16 (1.6%) would have an unacceptably high ratio, as many of the samples with a ratio greater than 1.9 had a ratio between 1.9 and 2.0 (Supporting Fig. 1). Samples stored for 16 years were notable for having the lowest median ratio, samples stored for 21, 12, and 8 years were similar, and samples stored for 7 years had the highest median ratio. Twelve-year-old samples most commonly had a satisfactory ratio, and there seemed to be a trend of decreasing number of samples with a high ratio with increasing sample age. However, applying a ratio of up to 2.0 as pure, only 7 samples stored for 7 years had a ratio > 2.0. A high ratio (> 1.9) can be caused by RNA contamination^29^, which is very likely here, as the QIAmp Mini spin columns co-purify RNA if it is present. RNase can be added during isolation to remove RNA^16^. In the present study, the aim was to investigate if it was at all possible to isolate DNA from the present samples. Amplification and sequencing would be necessary to thoroughly investigate contamination and the suitability of isolated DNA for further analyses. Additionally, it would be interesting to assess the presence of any bacterial growth in the samples that could affect the quantity and quality of DNA.

Furthermore, the choice of solvent can also affect the DNA absorption^29^, and we used an elution buffer with a pH of 9.0^30^, which increases the A260/280 ratio by 0.2–0.3 compared to the “true” A260/280 ratio^29^. Overall, 152 (15.0%) had a ratio below 1.7, which might indicate the presence of protein or other contaminants, since these absorb strongly around 280 nm^29^. As there was no distinct pattern related to sample year, the low ratio in some samples could be due to sample volume, quality, or isolation method.

Some samples, regardless of the storage duration, showed either low DNA quantities or A260/280 ratios greater than 1.9 or less than 1.7. We observed no specific reason for the occurrence of the outliers but clotting could potentially still be a factor for an individual sample. To further investigate what causes low ratio, the 260/230 ratio could be analysed, or other isolation methods could be tested.

DNA fragmentation is important to assess, as intact DNA fragments are required for high-quality sequencing. Long fragments are more likely to overlap and contain sequences of interest, depending on the project aims, while short fragments are more likely to disrupt the sequence of interest^31^. Different kinds of sequencing require different quality and concentration parameters. For example, sequences > 10 kbp can be generated from long-read sequencing^32^, but to apply this method (e.g., long-read sequencing from Pacific Biosciences), the DNA must have a A260/280 ratio of 1.8–2.0 and a fragment size around 50 kbp^33^. For short-read sequencing, such as next-generation sequencing, the DNA is amplified and fragmented into smaller pieces around 250–800 bp and then sequenced in parallel. For some specific regions of interest, shorter fragment length DNA can be used. In this study, we were most interested in the possibility of isolating DNA with an intact region of interest rather than how long the fragments are, since the region of interest can be amplified^32^. As most of our samples were pure (based on A260/280 ratio) and of satisfactory quality (based on DIN), it is very likely that they can be used in further sequencing analyses. These samples need to be further analysed for specific regions of interest and in different downstream applications.

Supporting Fig. 2 shows that the DIN is not solely determined by fragment size, since some samples of size > 50 000 bp had a DIN below 7. Thus, other quality parameters, such as concentration and A260/280 ratio, should also be considered for individual samples. Nevertheless, we observed a pattern of decreased DIN with fragment size below 30,000 bp. As all groups had a median DIN greater than 7, many samples in our cohort contain high molecular weight DNA. Fragment size was significantly different across the years, but the difference in size between newer and older samples was not so great to assume that isolating DNA from newer samples produces DNA with longer fragments. Most samples with a fragment size between 40,000 and 60,000 bp were observed in samples stored for 12 years. However, 17 samples from the TapeStation were excluded prior to analysis due to an error with the analysed chip, resulting in fewer results from samples stored for 16 and 21 years. Therefore, comparing the number of samples of a specific fragment size based on year may be misleading. Additional samples from each sample group need to be analysed to further explore this association.

The DNA yield depends on the type of sample source and amount of material. Exceeding the suggested amount of starting material can lead to significantly lower yields than expected. A sample of lymphocytes is described to not exceed 5 × 10e6 cells^16^. Due to the low volumes of the present samples, the number of cells should not exceed 5 × 10e6. During optimization, we found that we were able to load the columns twice if the volume of starting material exceeded 250 µL, to minimize wastage and optimize yield. From these samples we obtained significantly higher yield per 1 mL of blood cell sample compared to samples with lowe than 250 µL sample volume. It is necessary to evaluate this DNA further and determine if there has been any effect on the purity and quality of the DNA or if it is just easier to isolate higher yield from larger sample volumes.

The DNA yield was expected to decrease for older samples and as the samples had been heavily mistreated. However, we did not observe a decrease in DNA yield over time, in contrast to Chen et al.^13^. Even though the sample volumes differed due to clots and dried-in samples were diluted using PBS, which may introduce some error, it is interesting to note that the lowest DNA concentrations were found in samples stored for 21, 16, and 12 years (Fig. 1A and Table 1a). This could indicate that isolating DNA from older samples carries a greater risk of a low DNA concentration. Overall, assuming blood samples are stored within the desired temperature range at stable conditions, DNA can be extracted from study samples after prolonged storage at – 20°.

The age of a study participant may influence the number of white blood cells available in the sample^9,34^. However, that applies more to adults and later life and is not relevant for the present study. With age progression, the quantity of isolated DNA can decline due to a reduction in the number of leukocytes and lymphocytes^35^. The samples in the present study included only samples from children aged 2–15 years, and we therefore expect no influence on the quantity of DNA according to age.

One limitation of this study was the limited and varied sample volumes. QIAamp DNA Mini Blood Kits from Qiagen were the most cost effective and flexible method for this study, as the equipment was already available to us. Other methods of DNA isolation, such as manually extracting DNA using a salting out method^1^ or using phenol/chloroform extraction^36^, are more time consuming when isolating DNA from a large number of samples and require much larger sample volumes. The Qiagen kit may not be the best to use if samples are very clotted. A similar study^25^ found that the Quick-DNA Miniprep Plus Kit (Zymo Research, Irvine, CA) was most time efficient and produced the highest DNA yields when testing four different kits for extracting DNA from old blood samples, although the Qiagen DNeasy Blood and Tissue Kit resulted in highest quality DNA. These results for only ten samples cannot be fully applied to our study, but it shows how the choice of method affects the outcome. Another limitation is that we did not perform TapeStation measurements to assess DNA integrity for all 1012 samples. However, the large subset of 270 samples provides confidence in the DNA integrity of the entire cohort of isolated DNA from long-term stored blood cell samples. A more comprehensive analysis would include electrophoresis, fluorometry, quantitative PCR, or a long-range PCR. Automated techniques such as TapeStation represent a form of automated electrophoresis where the software compares the result from the sample to a genomic DNA ladder and presents sizes and quantifications as charts and numbers. The advantages of using such a method are that both preparation and analysis are less time consuming and require a much smaller sample volume^37–39^.

The strength of this project is that the samples were obtained as part of the DiPiS study, in which sample and data collection were performed according to protocol. The quality of the samples in this study prior to freezing was unknown, unlike samples in similar studies. In the DiPiS study, many of the samples were taken at home by the families and not by trained personnel, sent by post to the laboratory and potentially mishandled during storage. To our best knowledge, no other study has investigated DNA isolation from heavily mishandled samples that have been stored for long periods. Another study evaluating the effects of repeated freezing and thawing peripheral blood on DNA yield and integrity detected decrease in yield but no degradation of DNA^40^. Removing plasma from the blood samples in the DiPiS study did not remove any of the pelleted cells, and it is unknown if removing the plasma has a positive or a negative effect on the sample during long-term storage. Future studies will involve testing these samples by genotyping and comparing the results with previous SNP genotyping and Next Generation Sequencing from before storage.

This study critically addresses the question that many research labs have: “can I perform modern analyses like Next Generation Sequencing on my historical, old sample cohorts?” The answer is, largely, yes.

This study suggests that it is possible to isolate DNA of satisfactory quantity and quality after long-term storage and mistreatment through freeze–thaw cycles of blood cell samples stored separately from their corresponding plasma in capillary tubes at − 20°. Despite the limitations of this study, we found no evidence that DNA integrity worsened by mistreating the samples and with increasing sample age. Whole blood samples and blood cell samples in long-term storage under less-than-optimal conditions can be used in downstream analyses.

## Supplementary Information

Supplementary Information.

## Data Availability

All required data will be available with the corresponding author upon reasonable request if also conforming to existing data protection regulation.

## Abbreviations

DIN: DNA integrity number
DiPiS: Diabetes Prediction in Skåne
