# Supplementary material for: Feasibility of extracting usable DNA from blood samples stored up to 21 years in the DiPiS study
Source: Sci Rep. 2025 Jul 15;15:25637. doi: 10.1038/s41598-025-08257-y (PMC12264131; doi:10.1038/s41598-025-08257-y)
Supplement: Supplementary file 1 — Supplementary Information. [file 41598_2025_8257_MOESM1_ESM.docx]

# Supporting information

**Supporting Table 1. Characteristics of DNA concentration and A260/280 stratified by storage duration in years.** The ratio is grouped into low ratio (<1.7), ratio within satisfactory range (1.7-1.9) and high ratio (>1.9). Concentrations and A260/280 ratios were measured using NanoQuant Plate in a Tecan Infinite 200 Pro reader.

|  |  |  | **Storage duration of blood cell samples (year)** | | | | |
| --- | --- | --- | --- | --- | --- | --- | --- |
| **A260/280** | **Concentration (ng/µL)** | **n** | **21** | **16** | **12** | **8** | **7** |
| **Low** | **< 10** | 1 | 1 (100%) | 0 (0%) | 0 (0%) | 0 (0%) | 0 (0%) |
|  | **10 - 20** | 10 | 1 (10.0%) | 5 (50.0%) | 0 (0%) | 1 (10.0%) | 3 (30.0%) |
|  | **20 - 79** | **102** | 12 (11.8%) | 29 (28.4%) | 16 (15.7%) | 18 (17.6%) | 27 (26.5%) |
|  | **80 - 119** | **23** | 11 (47.8%) | 1 (4.3%) | 1 (4.3%) | 5 (21.7%) | 5 (21.7%) |
|  | **120 - 150** | **11** | 5 (45.5%) | 0 (0%) | 3 (27.3%) | 2 (18.2%) | 1 (9.1%) |
|  | **> 150** | **5** | 2 (40.0%) | 3 (60.0%) | 0 (0%) | 0 (0%) | 0 (0%) |
| **Satisfactory** | **< 10** | **2** | 1 (50.0%) | 0 (0%) | 1 (50.0%) | 0 (0%) | 0 (0%) |
|  | **10 - 20** | **17** | 2 (11.8%) | 5 (29.4%) | 2 (11.8%) | 2 (11.8%) | 6 (35.3%) |
|  | **20 - 79** | **454** | 91 (20.0%) | 116 (25.6%) | 70 (15.4%) | 101 (22.2%) | 76 (16.7%) |
|  | **80 - 119** | **223** | 48 (21.5%) | 24 (10.8%) | 71 (31.8%) | 37 (16.6%) | 43 (19.3%) |
|  | **120 - 150** | **56** | 12 (21.4%) | 6 (10.7%) | 12 (21.4%) | 8 (14.3%) | 18 (32.1%) |
|  | **> 150** | **33** | 7 (21.2%) | 2 (6.1%) | 14 (42.4%) | 5 (15.2%) | 5 (15.2%) |
| **High** | **< 10** | **2** | 1 (50.0%) | 1 (50.0%) | 0 (0%) | 0 (0%) | 0 (0%) |
|  | **10 - 20** | **2** | 0 (0%) | 0 (0%) | 0 (0%) | 1 (50.0%) | 1 (50.0%) |
|  | **20 - 79** | **56** | 6 (10.7%) | 6 (10.7%) | 8 (14.3%) | 14 (25.0%) | 22 (39.3%) |
|  | **80 - 119** | **11** | 0 (0%) | 2 (18.2%) | 2 (18.2%) | 2 (18.2%) | 5 (45.5%) |
|  | **120 - 150** | **4** | 0 (0%) | 0 (0%) | 0 (0%) | 4 (100%) | 0 (0%) |
|  | **> 150** | **NA** | NA | NA | NA | NA | NA |


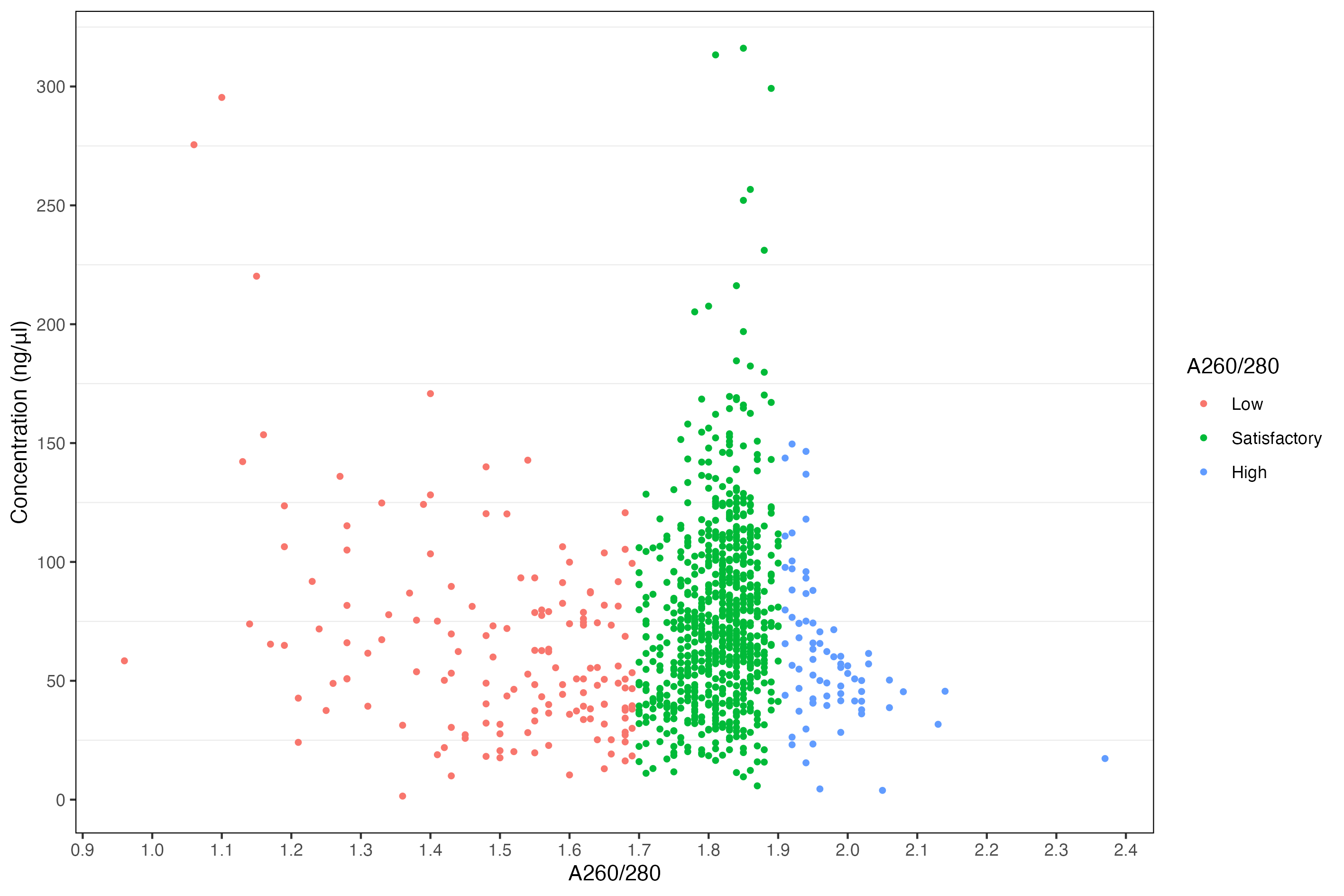


**Supporting Figure 1.** **DNA concentration (ng/μL) and A260/280** **ratio of 1012 DNA samples in the DiPiS study.** The colours (red, green, blue) corresponds to a low ratio (<1.7), ratio within satisfactory range (1.7-1.9) and high ratio (>1.9), respectively.


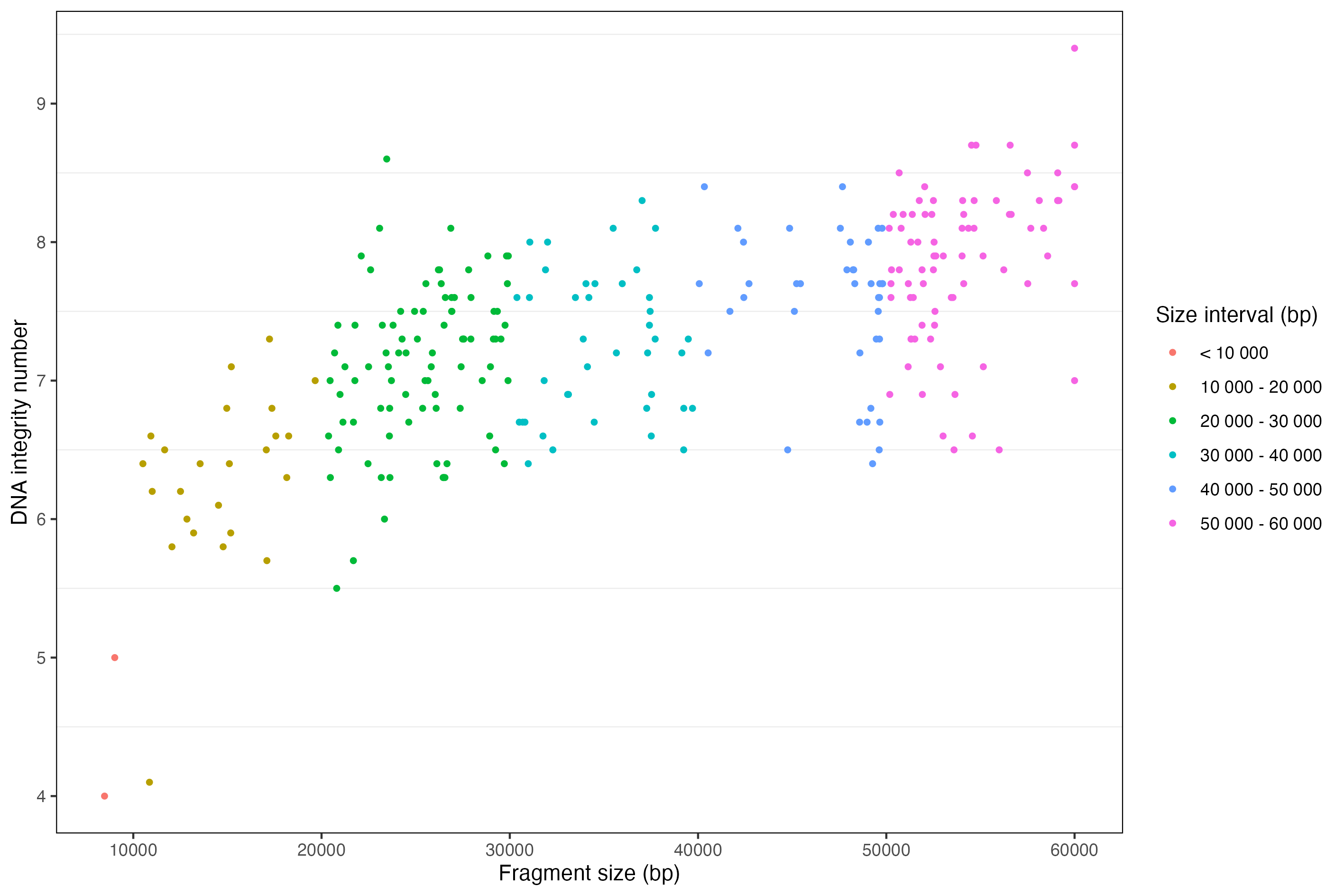


**Supporting Figure 2. DNA integrity number (DIN) and fragment size measured in base pairs (bp) of 270 DNA samples in the DiPiS study and colored by fragment size.**


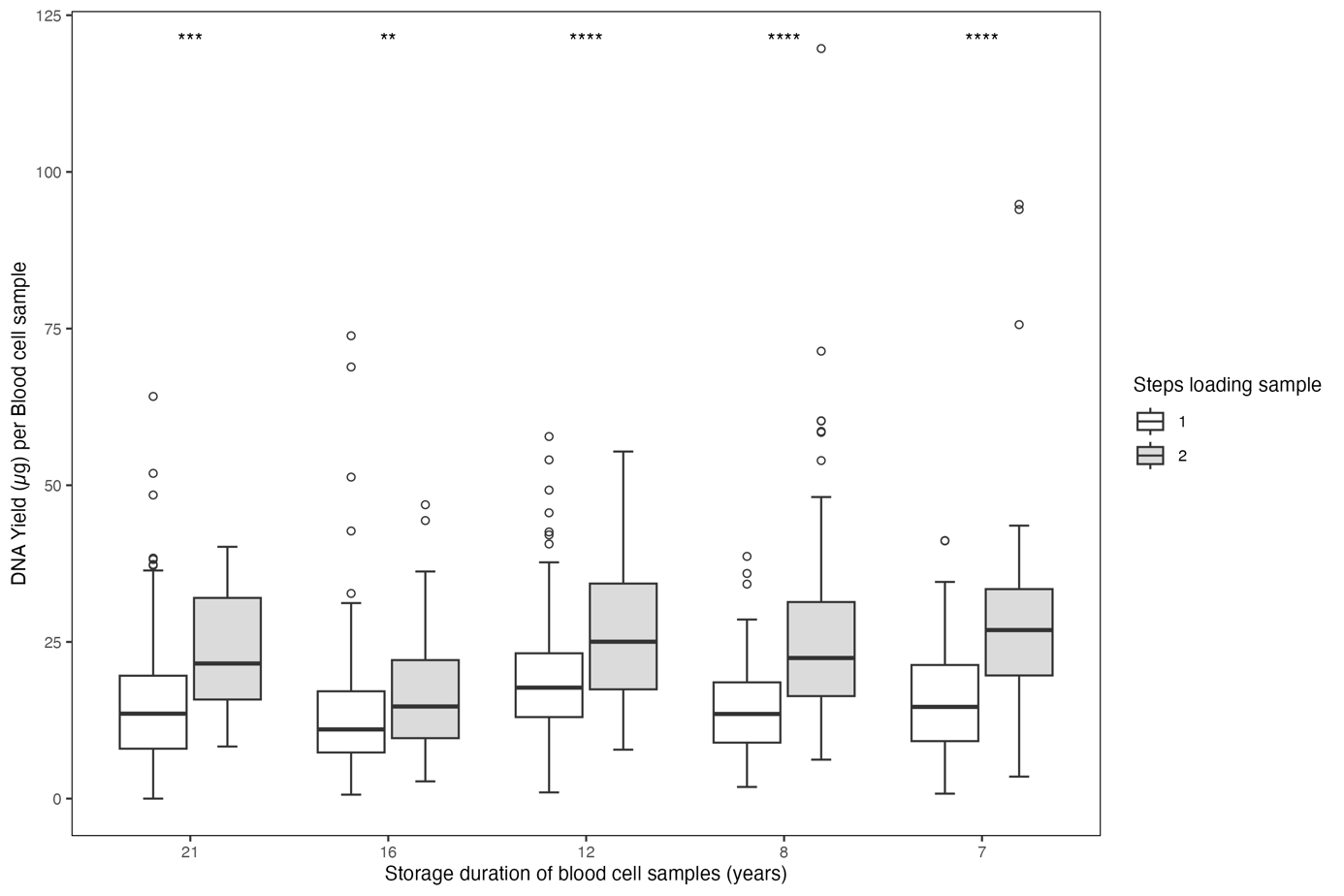


**Supporting Figure 3. DNA yield (µg) per 1 mL of blood cell sample compared between samples loaded once (< 250 µL) or twice (> 250** **µL) blood cell sample.** Range and distribution of DNA yields per 1 mL of blood cells isolated from frozen blood cell samples stored over 7-21 years at -20°C during which they have been heavily mistreated. Samples loaded twice were found to have a higher yield throughout the dataset. DNA yields were calculated from concentrations measured by NanoQuant Plate in a Tecan Infinite 200 Pro reader. Wilcoxon’s test was used to estimate the significance of differences between groups.

**Members of the DiPiS study group:**

A. Ramelius^1^, C. Andersson^1^, R. Bennet^1^, M. Ask^1^, J. Bremer^1^, C. Brundin^1^, C. Cilio^1^, H. Elding Larsson^1^, C. Hansson^1^, G. Hansson^1^, S. Ivarsson^1^, B. Jonsdottir^1^, I. Jonsson^1^, B. Lindberg^1^, B. Lernmark^1^, Å. Lernmark^1^, J. Melin^1^, M. Lundgren^1,2^, A. Carlsson^1^, E. Cedervall^3^, B. Jönsson^4^, K. Larsson^2^ and J. Neiderud^5^

^1^ Department of Clinical Sciences, Lund University, Sweden.

^2^ Department of Paediatrics, Kristianstad Hospital, Kristianstad, Sweden

^3^ Department of Paediatrics, Ängelholm Hospital, Sweden

^4^ Department of Paediatrics, Ystad Hospital, Sweden

^5^ Department of Paediatrics, Helsingborg Hospital, Sweden
